# Supplementary material for: Use of Raman and Raman optical activity to extract atomistic details of saccharides in aqueous solution
Source: PLoS Comput Biol. 2022 Jan 20;18(1):e1009678. doi: 10.1371/journal.pcbi.1009678 (PMC8806073; doi:10.1371/journal.pcbi.1009678)
Supplement: Attachment — Submitted filename: rebuttal.pdf [file pcbi.1009678.s003.pdf]

Reply to Reviewers - Use of Raman and Raman optical activity to extract atomistic details of saccharides in aqueous solution

Vladimír Palivec,† Christian Johannessen,‡ Jakub Kaminský,† and Hector Martinez-Seara*,†

†*Institute of Organic Chemistry and Biochemistry, Czech Academy of Sciences, Prague, Czech Republic*

‡*Department of Chemistry, University of Antwerp, Antwerp, Belgium*

E-mail: hseara@gmail.com

Reviewer 1

In their manuscript, the authors present their methodology of improved calculations of Raman, and Raman optical activity spectra of carbohydrates in aqueous solution and analysis of their ring puckering conformations, anomeric ratios, or glycosidic bonds, extended for a bigger systems, e.g. disaccharides and trisaccharides. There are limited tools to study carbohydrate structure in a native aqueous solutions. ROA/Raman tend to be very useful in this field, showing great potential, however analysis based on theoretical calculations are challenging. Their work is very laborious and impressive. Combination of Raman/ROA experiments with MD and QM based calculations, together with modern approach of partial optimization of snapshots, scaling functions or best fit procedure, gave great results of the similarity index of the experimental and simulated spectra, and extensive information

of the carbohydrate structure. Although this work is suitable for publication in the PLOS Computational Biology, several questions and concerns arises after reading, that are listed below.

R1Q1

Question

Please describe precisely, point by point what is the novelty of present manuscript compared to former paper from Palivec et al. PCCP.,2020, 22, 1983 (10.1039/c9cp05682c). My first impression was that it is to some point extend of the former paper to di- and trisaccharides. Please clarify in the manuscript, what is new here and what is based on the previous paper.

Answer

Our former work (DOI:10.1039/c9cp05682c) was dedicated to developing an optimized simulation protocol to obtain Raman/ROA spectra of saccharides in aqueous solution. On a reduced set of six monosaccharides, we tested various solvation approaches, optimization schemes, DFT functionals, basis sets, frequency scaling approaches, and other methodological details. As a results we proposed a simulation protocol that offers a quality Raman/ROA spectra that is also extremely cost-efficient to simulate. The aim of current work is to show how our previously developed protocol can be used to access to a plethora of structural features of saccharides in solution. We now show that we when combining the experimental spectra with our protocol we can obtain various features of saccharides in solution (puckering, glycosidic bond, mixtures, larger saccharides) which was not the point and most were not even tackled on our former work. In fact, our present work shows that our previous protocol with just small fine tuning can tackle significantly more complex tasks that those we used for its development, including state of the art cases for ROA/Raman techniques such as trisaccharides. To sum up, our former work focuses on technicalities of simulating Raman/ROA spectra, while the current work focuses of using the developed protocol to interpret experi-

mental data and access various structural features of saccharides in solution. These are two completely different aspects, although we obviously agree with the reviewer that without our previous methodological work, this work will not be possible and we certainly build on our previously developed technique to show how powerful it is.

We never hide this fact, and our previous work is heavily cited in the present one to make clear its origin. Still, to avoid any misinterpretation, we have added some clarifying text to the manuscript that we believe emphasizes the differences with our previous work.

R1Q2

Question

To understand properly all methods used here, I read the authors' previous work (Palivec et al. PCCP.,2020, 22, 1983), together with the Reply to Reviewers that was added as the SI. After careful reading, I still have concerns about scaling of calculated intensities. In the previous paper to which the reader is referred, you mentioned "The average magnitudes of ROA and Raman intensities are experimentally related (I(ROA) I(Raman)x10^{-4}), however, we optimize both spectral intensities separately. As a result, the ratio is not necessarily preserved during the optimization", Is it the case also in this paper? If yes it should be mentioned somewhere, and discussed, especially because all simulated and experimental ROA/Raman spectra are presented here in some puzzling arbitrary units, where ROA and Raman intensities are comparable, although one can expect rather 10^{-4} ratio of ROA/Raman intensities. In my opinion it will be puzzling for future readers. I have a problem with it because you are losing information about CID ratios. As we all know, CID ratios are inherently associated with ROA, and their calculation and comparison with experiment can improve the reliability of structural conclusions (Polavarapu, CHIRALITY 26:539–552 (2014)). At least I would suggest that the original ROA/Raman ratios of both experimental and simulated spectra should be preserved during all the scaling, and minimization of the cost function. I'm curious how would it change the final results?

Answer

The reviewer is concerned about scaling of calculated Raman/ROA spectral intensities to match experimental ones using two different intensity scaling factors and asks how would this change our results. Yes, we are using the simulation method developed in our previous work. Therefore we are using two different scaling factors, which does not preserve the CID ratios as was already explained and thoroughly discussed in the methodological paper. Firstly, we would like to stress that our methodology is completely agnostic to absolute intensities of Raman/ROA spectra. Therefore, our final structure prediction results would remain completely unchanged. This is because their evaluation is based upon optimizing overlap integral values, which are independent on individual intensities. Therefore, the only thing that would change with using same scaling factors is the visual comparison of Raman/ROA spectra.

Moreover, we would like to stress that the CID shape remains unchanged during the whole process, up to a constant. This is because we scale Raman/ROA with two factors(ρ_{Raman}, ρ_{ROA}) and so

$$CID = \frac{I_{ROA} \cdot \rho_{ROA}}{I_{Raman} \cdot \rho_{Raman}} = CID \frac{\rho_{ROA}}{\rho_{Raman}}. \quad (1)$$

Therefore, still, our spectra can be used to produce CID which can be compared to experimentally observed CID. Lastly, we checked the difference between separate scaling factors and single scaling factor on three moieties and the average difference is negligible, i.e., 2.7%. To summarize, our two factor scaling have no effect whatsoever on our structure predictions and the difference as compared to a single scaling factor is negligible, therefore, visually there would be essentially no difference upon switching to a single scaling factor(up to 2.7% change in intensities). Altogether, change to a single scaling factor would effectively have no effect on current manuscript at all.

Nevertheless, we agree that using two scaling factors and not preserving CID ratios is an important part of our approach and therefore we are now discussing it in the manuscript.

Moreover, we agree that using ideas that are discussed in (Polavarapu, CHIRALITY 26:539–552 (2014)) are valuable, e.g., the concept of robustness, and could be used to further improve the reliability of structural predictions of our methodology.

R1Q3

Question

About bibliography. I would suggest to enrich the bibliography with the latest papers on ROA of trisaccharides, or polysaccharides e.g. 10.1039/C9CP00472F, or 10.1016/j.saa.2018.08.017, where in the latter, raffinose ROA spectrum is discussed.

Answer

The discussion now includes these works as they certainly justify the interest of this work to cover larger saccharides.

Reviewer 2

R2Q1

Question

This manuscript reports a detailed structural study of six model monosaccharides in water using Raman, ROA and sometimes NMR data, coupled with a combined molecular dynamic (MD) and quantum mechanics (QM) approach. One well-known challenge in interpreting the VOA spectra observed is how to properly account for water solvation. In this study, the authors accounted for the effects of water on the conformationally flexible sugar molecules by using free energy surface profiles (FES) generated in the respective MD simulations to identify possible conformations. Using a previously developed hybrid simulation approach (ref. 10), the authors took MD snapshots of the central molecule together

with surrounding water molecules (3 A cutoff) and calculated their Raman and ROA spectra by treating water molecules at the MM level and the solute at the QM level using the ONIOM method. The authors clearly demonstrated that the above approach, in combination with Raman, ROA and NMR experiments, is a viable method to gain insights into structural features of sugars in solutions. In particular, their simulations take advantage of the sensitivity of Raman and ROA to small structural changes in rotation around the glycosidic bonds and in the puckering of the sugar rings, allowing significant insights into structural features of sugars in solution. The manuscript is clear and well written. However, some additional solvation references should be added, for example, a review on the evidence of the long-lived solvent-water clusters, related to the induced water VCD features (https://doi.org/10.3389/fchem.2016.00009) and a recent example using this simplified model for IR/VCD and Raman/ROA (https://doi.org/10.1002/cphc.201800309). Also, an exciting work of AIMD ROA (https://doi.org/10.1021/acs.jpclett.7b01616) and a recent QM/MM simulation of IR/VCD and Raman/ROA of pantolactone (https://dx.doi.org/10.1021/acs.jpcb.0c[illegible] should be cited.

Answer

We thank the review for the suggestions and we agree. The citations have been added and are now discussed in the introduction.

R2Q2

Question

There are some words used whose meaning is ambiguous. For example, the authors used "mobile" often in the text. "Unfortunately, many available structure characterization techniques are inadequate when applied to mobile molecules such as saccharides". All molecules are mobile in solution. I think the authors want to emphasize "flexibility" of saccharides and should change the wording accordingly.

Answer

We agree and we changed the text accordingly.

R2Q3

Question

"ROA is a Raman-based spectroscopic method where the difference in right/left circularly polarised light spectra is recorded." A more precise definition of ROA should be provided.

Answer

We change the text to provide better and more precise definition of ROA:

"ROA is a Raman-based spectroscopic method where the difference in Raman spectra when using right(I_R)/left(I_L) circularly polarised light spectra is recorded, i.e., $I_{ROA}=I_R-I_L$."

R2Q4

Question

"The advantage of Raman/ROA techniques when studying saccharides is a transparent vibrational spectral region between 75 and 3100 cm^{-1}," Does this refer to water, the solvent, which has weak Raman in the region?

Answer

Yes, but also to the transparency of the optics used. Since the Raman effect is a small shift away from the incident photons, optics that are transparent in the visual range can be used even though we are probing vibrational frequencies. See below for limitations in IR.

R2Q5

Question

Can one apply the same approach to VCD spectra of chiral molecules in water? How well will it work? Can the authors make some comments on this aspect?

Answer

The computational approach can easily be applied to VCD(as documented in ref. 62). The problem with VCD on carbohydrates is experimental. While application of Raman/ROA on carbohydrates result in strong signals, IR/VCD on this group of molecules is very weak, making them less appealing targets for VCD analysis(as can be seen, e.g., in Chirality 2008, 20, 446; JACS 2004, 126, 9496; Org. Biomol. Chem., 2007, 5, 1104; Carbohydr. Res. 2004, 339, 2713). Combine that with water being a strong absorber of IR radiation and limitations to range due to limitations to optical transparency, IR/VCD on carbohydrates would be limited to the range 1000-1500 cm^{-1}. This is severely limiting the amount of information that can be compared to the predicted spectra, and therefore Raman/ROA is the usual target when characterizing saccharides in solution.

R2Q6

Question

In conclusion, this is an interesting and well-performed study, which fully deserves publication in PLOS Computational Biology pending the modifications suggested above.

Answer

We thank the reviewer for his kind words, and we hope that the changes and clarifications provided above are satisfactory.
