# Supplementary material for: Use of Raman and Raman optical activity to extract atomistic details of saccharides in aqueous solution
Source: PLoS Comput Biol. 2022 Jan 20;18(1):e1009678. doi: 10.1371/journal.pcbi.1009678 (PMC8806073; doi:10.1371/journal.pcbi.1009678)
Supplement: Attachment — Submitted filename: PLOS_ROA_report.docx [file pcbi.1009678.s002.docx]

This manuscript reports a detailed structural study of six model monosaccharides in water using Raman, ROA and sometimes NMR data, coupled with a combined molecular dynamic (MD) and quantum mechanics (QM) approach. One well-known challenge in interpreting the VOA spectra observed is how to properly account for water solvation. In this study, the authors accounted for the effects of water on the conformationally flexible sugar molecules by using free energy surface profiles (FES) generated in the respective MD simulations to identify possible conformations. Using a previously developed hybrid simulation approach (ref. 10), the authors took MD snapshots of the central molecule together with surrounding water molecules (3 A cutoff) and calculated their Raman and ROA spectra by treating water molecules at the MM level and the solute at the QM level using the ONIOM method. The authors clearly demonstrated that the above approach, in combination with Raman, ROA and NMR experiments, is a viable method to gain insights into structural features of sugars in solutions. In particular, their simulations take advantage of the sensitivity of Raman and ROA to small structural changes in rotation around the glycosidic bonds and in the puckering of the sugar rings, allowing significant insights into structural features of sugars in solution.

The manuscript is clear and well written. However, some additional solvation references should be added, for example, a review on the evidence of the long-lived solvent-water clusters, related to the induced water VCD features (https://doi.org/10.3389/fchem.2016.00009) and a recent example using this simplified model for IR/VCD and Raman/ROA (https://doi.org/10.1002/cphc.201800309). Also, an exciting work of AIMD ROA (https://doi.org/10.1021/acs.jpclett.7b01616) and a recent QM/MM simulation of IR/VCD and Raman/ROA of pantolactone (https://dx.doi.org/10.1021/acs.jpcb.0c01483) should be cited.

There are some words used whose meaning is ambiguous. For example, the authors used “mobile” often in the text. “Unfortunately, many available structure characterization techniques are inadequate when applied to **mobile** molecules such as saccharides”. All molecules are mobile in solution. I think the authors want to emphasize “flexibility” of saccharides and should change the wording accordingly.

“ROA is a Raman-based spectroscopic method where the difference in right/left circularly polarised light spectra is recorded.” A more precise definition of ROA should be provided.

“The advantage of Raman/ROA techniques when studying saccharides is a transparent vibrational spectral region between 75 and 3100 cm-1,” Does this refer to water, the solvent, which has weak Raman in the region?

Can one apply the same approach to VCD spectra of chiral molecules in water? How well will it work? Can the authors make some comments on this aspect?

In conclusion, this is an interesting and well-performed study, which fully deserves publication in PLOS Computational Biology pending the modifications suggested above.
